# Green Fluorescent Protein and Phase-Contrast Image Fusion via Generative Adversarial Networks

**DOI:** 10.1155/2019/5450373

**Published:** 2019-12-04

**Authors:** Wei Tang, Yu Liu, Chao Zhang, Juan Cheng, Hu Peng, Xun Chen

**Affiliations:** ^1^Department of Biomedical Engineering, Hefei University of Technology, Hefei 230009, China; ^2^Department of Electronic Science and Technology, University of Science and Technology of China, Hefei 230026, China

## Abstract

In the field of cell and molecular biology, green fluorescent protein (GFP) images provide functional information embodying the molecular distribution of biological cells while phase-contrast images maintain structural information with high resolution. Fusion of GFP and phase-contrast images is of high significance to the study of subcellular localization, protein functional analysis, and genetic expression. This paper proposes a novel algorithm to fuse these two types of biological images via generative adversarial networks (GANs) by carefully taking their own characteristics into account. The fusion problem is modelled as an adversarial game between a generator and a discriminator. The generator aims to create a fused image that well extracts the functional information from the GFP image and the structural information from the phase-contrast image at the same time. The target of the discriminator is to further improve the overall similarity between the fused image and the phase-contrast image. Experimental results demonstrate that the proposed method can outperform several representative and state-of-the-art image fusion methods in terms of both visual quality and objective evaluation.

## 1. Introduction

In the field of cell and molecular biology, fluorescent imaging and phase-contrast imaging are two representative imaging approaches. As a widely used tool in fluorescent imaging, green fluorescent protein (GFP) displays bright green fluorescence when exposed to light in the range of blue to ultraviolet. The GFP image contains functional information related to the molecular distribution of biological cells but has very low spatial resolution. Phase-contrast imaging is an optical microscopy technique that visualizes phase shifts through converting it to variation of amplitude or contrast in the image. The phase-contrast image provides structural information with high resolution. Fusion of GFP image and phase-contrast image is of great significance to the localization of subcellular structure, the functional analysis of protein, and the expression of gene [1].

In recent years, a variety of image fusion methods have been proposed. Generally, existing image fusion algorithms mainly consist of three steps: image transform, fusion, and inverse transform [2]. The representative fusion methods include multiscale transform-based ones [3–8], sparse representation-based ones [9–13], spatial domain-based ones [14–17], hybrid transform-based ones [18–21], etc. In most of the existing image fusion methods, the role of each input image is equivalent in terms of the fusion system, which means that the input images generally undergo identical transforms and uniform fusion rules. However, for the problem of GFP and phase-contrast image fusion, considering that the input images vary significantly from each other, different roles can be assigned to them in the fusion system by carefully addressing their own characteristics, which is likely to provide a more effective way to tackle this fusion issue.

**Figure 1:**
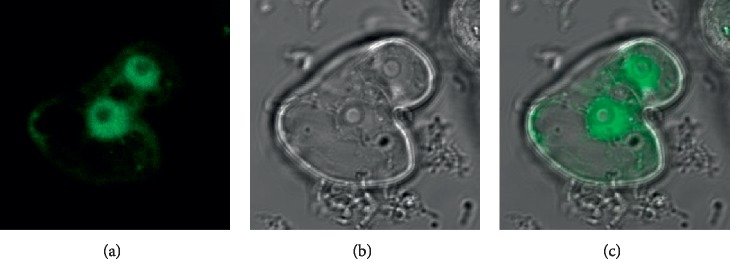
A pair of GFP and phase-contrast images and the fusion result obtained by the proposed method. (a) The GFP image. (b) The phase-contrast image. (c) The fused image of the proposed method.

In this paper, we propose a novel GFP and phase-contrast image fusion method based on generative adversarial networks (GANs). The fusion problem is modelled as an adversarial game between a generator and discriminator. The aim of the generator is to obtain a fused image that integrates the functional information from the GFP image together with the structural information from the phase-contrast image, while the discriminator further ensures the overall similarity between the fused image and the phase-contrast image. This adversarial process enables the fusion result to capture the complementary information from different input images as much as possible. An example of the proposed method is illustrated in Figure 1, where the input GFP and phase-contrast images are shown in Figures 1(a) and 1(b), respectively.  Figure 1(c) shows the fusion result obtained by the proposed method. By referring to the input images, it can be seen that our method achieves high performance in terms of the preservation of functional and structural information. The main contributions of this paper are summarized as follows:

1. We propose a deep learning- (DL-) based GFP and phase-contrast image fusion method via generative adversarial networks (GANs). To extract information from these two kinds of biological images adequately, the input images are treated differently in the proposed fusion model according to their own characteristics.
2. Extensive experiments on more than 140 pairs of input images demonstrate that the proposed method outperforms several representative image fusion methods in terms of both visual quality and objective evaluation.

The remainder of this paper is organized as follows.  Section 2 depicts some related works. In Section 3, the proposed GAN-based image fusion method is introduced in detail. The experimental results and discussions are given in Section 4. Finally, Section 5 concludes the paper.

## 2. Related Work and Motivations

### 2.1. GFP and Phase-Contrast Image Fusion

Fusion of GFP and phase-contrast images is conducive to the study of subcellular localization and functional properties of protein. In the past few years, several image fusion methods have been proposed to address this issue [22–24]. Li and Wang [22] proposed a NSCT-based GFP and phase-contrast image fusion method. In their method, the intensity components of input images are decomposed by NSCT and the obtained coefficients are then merged by a variable-weight fusion rule. In [23], Feng et al. introduced a fusion approach for GFP and phase-contrast images based on sharp frequency localization contourlet transform (SFL-CT). To fuse the decomposed coefficients, they designed a maximum region energy- (MRE-) based rule, a maximum absolute value- (MAV-) based rule, and a neighborhood consistency measurement- (NCM-) based rule to merge the approximation subbands, the finest detailed subbands, and other detailed subbands, respectively. Recently, Qiu et al. [24] presented a complex shearlet transform- (CST-) based method to fuse GFP and phase-contrast images. The high-frequency subbands are fused with the traditional absolute-maximum rule, while a Haar wavelet-based energy rule is introduced to merge low-frequency subbands.

It is worth noting that all of the above GFP and phase-contrast image fusion methods are based on conventional multiscale transforms. Moreover, the role of each input image is equivalent in these fusion methods, as they handle the GFP image (more precisely, its intensity component) and phase-contrast image in the same way.

### 2.2. Deep Learning-Based Image Fusion

In recent years, due to the high effectiveness and convenience in feature representation of deep learning (DL) models, DL-based study has emerged as a very active direction in the field of image fusion [25]. Many DL models such as stacked autoencoders (SAEs) and convolutional neural networks (CNNs) have been employed in a wide range of image fusion problems including remote sensing image fusion [26, 27], multifocus image fusion [28–30], multiexposure image fusion [31, 32], medical image fusion [33, 34], and infrared and visible image fusion [35–37]. In [26], Huang et al. firstly introduced deep learning into remote sensing image fusion by applying a sparse denoising autoencoder to characterize the nonlinear mapping between low- and high-resolution multispectral image patches. Liu et al. [28] proposed a CNN-based multifocus image fusion method in which a Siamese network is designed to simultaneously act as the roles of activity level measurement and fusion rule. In [31], Kalantari and Ramamoorthi introduced a learning-based multiexposure image fusion approach via CNN to model the complex deghosting process in dynamic scenes. Hermessi et al. [33] presented a CNN-based medical image fusion method which preextracts the shearlet features of source images as network input. Most recently, Ma et al. [35] introduced a novel generative adversarial network- (GAN-) based infrared and visible image fusion method by modelling the fusion problem as an adversarial game, aiming to preserve infrared intensities and visible details at the same time. This work demonstrates the high potential of the GAN models for multimodal image fusion.

### 2.3. Motivations of This Work

In this work, considering that the characteristics of the GFP image and the phase-contrast image are significantly different, unlike the exiting fusion methods on this issue introduced in Section 2.1, different roles are assigned to the input images for extracting information from them more effectively. To this end, and inspired by the great progress recently achieved in image fusion by deep learning, a GAN-based GFP and phase-contrast image fusion method is presented. We mainly adopt the GAN-based fusion scheme introduced in [35] due to its effectiveness and simplicity in multimodal image fusion, while carefully devising the loss functions according to the characteristics of the GFP and the phase-contrast images. To the best of our knowledge, this is the first time that a DL-based approach is used in the field of GFP and phase-contrast image fusion.

## 3. The Proposed Method

### 3.1. Overview

**Figure 2:**
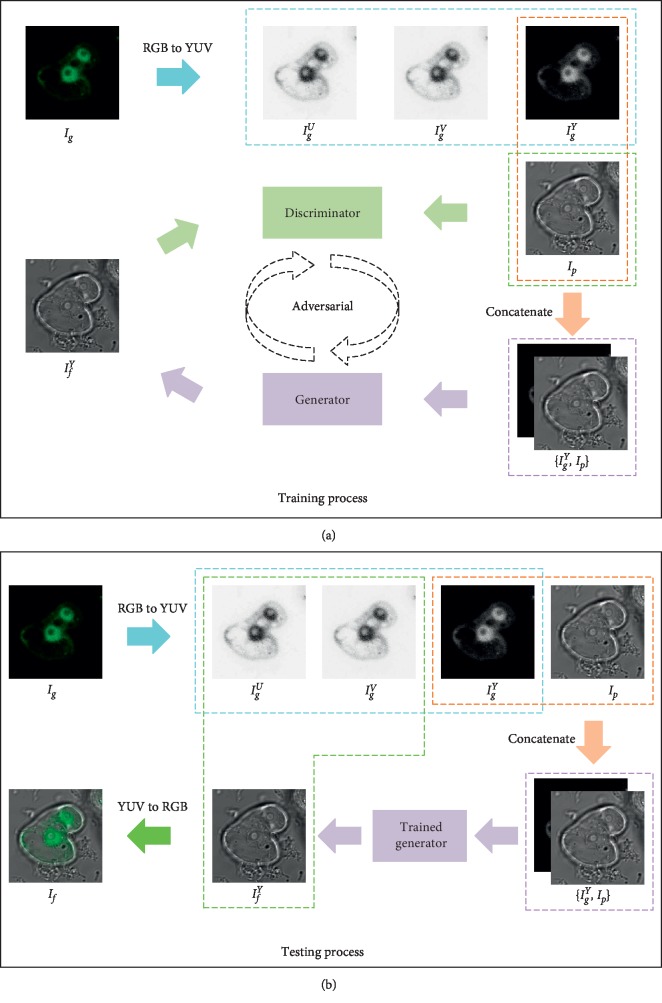
Schematic diagram of the proposed GAN for GFP image and phase-contrast image fusion.

Figure 2 shows the schematic diagram of the proposed GFP and phase-contrast image fusion method. The fusion issue is formulated as an adversarial problem to preserve the complementary information contained in the input images as much as possible. The GFP image is treated as an RGB color image in the fusion process. It is firstly converted into the YUV color space that can effectively separate the intensity or luminance component from the color image. Actually, this is a widely used approach in the field of functional and structural image fusion [6, 38].

During the training process, the GFP image *I*_*g*_ is converted into YUV color space to acquire the *Y*, *U*, and *V* components: *I*_*g*_^*Y*^, *I*_*g*_^*U*^, and *I*_*g*_^*V*^. Then, *I*_*g*_^*Y*^ and the phase-contrast image *I*_*p*_ are concatenated in the channel dimension to generate a two-channel map *I*_*c*_={*I*_*g*_^*Y*^, *I*_*p*_}, in which the first channel *I*_*c*_^1^=*I*_*g*_^*Y*^ and the second channel *I*_*c*_^2^=*I*_*p*_. Next, *I*_*c*_ is fed into the generator *G* and the output is termed as the intermediate fused image *I*_*f*_^*Y*^, which inclines to maintain the functional information of *I*_*g*_ and retain the structural information of *I*_*p*_. *I*_*f*_^*Y*^ and *I*_*p*_ are fed into the discriminator *D* to further ensure the overall similarity between them. In this way, adversarial game between *G* and *D* is founded.

During the testing process, *I*_*g*_^*Y*^ and *I*_*p*_ are concatenated in the channel dimension and then fed into the trained generator to obtain the intermediate fused image *I*_*f*_^*Y*^. The final fused image *I*_*f*_ is acquired by performing the inverse YUV conversion (i.e., YUV to RGB) over *I*_*f*_^*Y*^, *I*_*g*_^*U*^, and *I*_*g*_^*V*^.

### 3.2. Network Architecture

**Figure 3:**
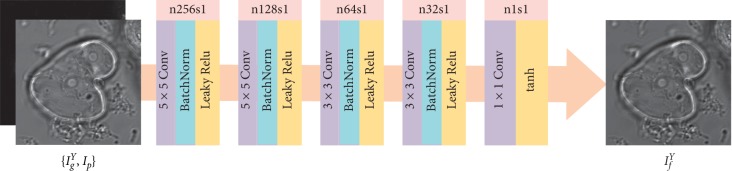
Network architecture of the generator. In each convolutional layer, there is no padding operation. During the training process, the input is image patches of size 112 × 112 pixels and the output is of size 100 × 100 pixels (see Section 3.4 for more details). During the testing process, the input is the entire images with 6 pixels padded in each direction to ensure that the output has the same size with the input images. For better visualization, we adopt the entire images as the input and output in this figure.

The network architecture of the generator is shown in Figure 3. The input of the generator is the concatenated *I*_*g*_^*Y*^ and *I*_*p*_, followed by a five-layer convolution network. The filters used in the first two layers, the next two layers, and the last layer are 5 × 5, 3 × 3, and 1 × 1, respectively. The symbol “n256s1” denotes the corresponding layer has 256 feature maps and the stride is 1, and so forth. In each convolutional layer, the stride is 1 and there is no padding operation. To preserve the details contained in the source images, the downsampling process is not adopted in each layer. Besides, to overcome the problems of vanishing gradient and data initialization sensitivity, batch normalization are employed in the first four layers. Leaky ReLU and tanh activation functions are used in the first four layers and the last layer, respectively. The output of *G* is the intermediate fused image *I*_*f*_^*Y*^.

**Figure 4:**
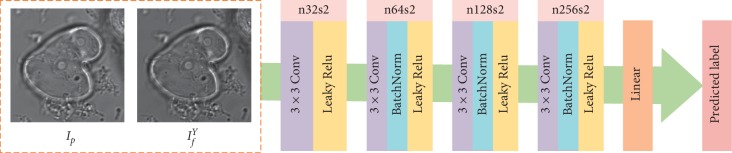
Network architecture of the discriminator. The discriminator only works in the training process. The input (either the central part of the phase-contrast image patch or the output of the generator) has the size of 100 × 100 pixels (see Section 3.4 for more details). For better visualization, we adopt the entire images as the input in this figure.

The network architecture of the discriminator is shown in Figure 4. The inputs of the discriminator are *I*_*p*_ and *I*_*f*_^*Y*^, followed by a five-layer convolution network where 3 × 3 filters are used in the first four layers with a stride of 2. The discriminator actually plays the role of a classifier. Batch normalization is employed in the second, third, and fourth layers, and the leaky ReLU activation function is used in the first four layers, and the last layer is a linear layer. The output of the discriminator is the predicted label (the dimension is one).

### 3.3. The Definition of the Loss Functions

The loss functions of our network are composed of two parts: the loss function of the generator *G* and the loss function of the discriminator *D*. To improve the quality of generated images and the stability of training process, they are designed based on the least squares generative adversarial networks (LSGANs) introduced by Mao et al. [39].

#### 3.3.1. The Loss Function of the Generator

The loss function of *G* is formulated as

(1) $$\begin{array}{c} {ℒ}_{G}=V_{\text{GAN}}\left(G\right)+\alpha {ℒ}_{C}, \end{array}$$

where *𝒱*_GAN_(*G*) and *ℒ*_*C*_ denote the adversarial loss between the generator and the discriminator and the content loss, respectively. The parameter *α* is used to control the balance between *𝒱*_GAN_(*G*) and *ℒ*_*C*_. The first term *𝒱*_GAN_(*G*) is defined as

(2) $$\begin{array}{c} V_{\text{GAN}}\left(G\right)=\frac{1}{N}\overset{N}{\underset{i=1}{\sum }}{\left(D\left(I_{f}^{Y\left(i\right)}\right)-c\right)}^{2}, \end{array}$$

where *N* is the number of training samples in a batch and *I*_*f*_^*Y*(*i*)^ denotes the fused image with *i* ∈ _*N*_. The parameter *c* is the value that the generator expects the discriminator to believe in terms of the fake data. The second term *ℒ*_*C*_ is formulated as

(3) $$\begin{array}{c} {ℒ}_{C}=\frac{1}{HW}{\left\|I_{f}^{Y}-I_{g}\right\|}_{F}^{2}+\beta \cdot \frac{1}{HW}{\left\|I_{f}^{Y}-I_{p}\right\|}_{F}^{2}+\gamma \cdot \text{SSIM}\left(I_{f}^{Y},I_{p}\right), \end{array}$$

where *H* and *W* indicate the height and width of the input images, respectively, ‖·‖_*F*_ denotes the matrix Frobenius norm, and SSIM represents the structural similarity operation [40]. The first term is designed to preserve the functional information of GFP image. The second term aims to extract the energy (represented by image intensity) of the phase-contrast image, and the third term is devised to maintain the structural information contained in the phase-contrast image. *β* and *γ* are trade-off parameters to balance these three terms.

#### 3.3.2. The Loss Function of the Discriminator

The information of *I*_*p*_ is incapable of being completely expressed only by its energy and structural information. For example, the texture details may not be fully extracted in this way. To further improve the overall similarity between *I*_*p*_ and *I*_*f*_^*Y*^, a discriminator *D* is introduced into the proposed framework. The loss function of *D* is formulated as

(4) $$\begin{array}{c} {ℒ}_{D}=\frac{1}{N}\overset{N}{\underset{i=1}{\sum }}{\left(D\left(I_{p}\right)-b\right)}^{2}+\frac{1}{N}\overset{N}{\underset{i=1}{\sum }}{\left(D\left(I_{f}^{Y}\right)-a\right)}^{2}, \end{array}$$

where *a* and *b* stand for the labels of *I*_*f*_^*Y*^ and *I*_*p*_, respectively.

### 3.4. Training Details

The popular GFP database, which is available at http://data.jic.ac.uk/Gfp/, released by the John Innes Centre [1] is employed as the training data in this work. The database contains 148 pairs of registered GFP and phase-contrast images of size 358 × 358 pixels that focus on the *Arabidopsis thaliana* cells.

In order to obtain sufficient data for network training, each input image is cropped into a large number of patches of the same size 112 × 112 pixels. The stride for cropping is set to 12. As a result, we totally acquire 65268 pairs of GFP and phase-contrast image patches, and the range of each patch is normalized to [−1,1]. In each iteration during training, the input of the generator contains *n* pairs of input image patches (i.e., the batch size is *n*), and the output intermediate fused patches and the phase-contrast patches (the central part of size 100 × 100 pixels) are employed as the input of the discriminator. Moreover, in each iteration, the discriminator is firstly trained *m* times (i.e., the training step is *m*) using the Adam optimizer [41] and then the generator.  Algorithm 1 summarizes the procedure of network training.

In our experiments, the parameters for training are set as follows. The batch size *n* and the number of epochs are set to 32 and 10, respectively. Accordingly, the number of training iterations is 65268 × 10/32 ≈ 20396. The training step of the discriminator *m* is fixed as 2, and the learning rate is set to 10^−4^. For easier training, as suggested in [35], soft labels are adopted for *a*, *b*, and *c*. That is, they are set to random numbers rather than specific ones. The label *a* of *I*_*f*_^*Y*^ and the label *b* of *I*_*p*_ are with the ranges of 0 to 0.3 and 0.7 to 1.2, respectively. The label *c* of *I*_*f*_^*Y*^ ranges from 0.7 to 1.2.

## 4. Experiments

### 4.1. Experimental Settings

#### 4.1.1. Testing Images

Considering that the proposed method is an *unsupervised* approach (there is no ground truth fused images for training), all the 148 pairs of images used for training in the GFP database [1] (as mentioned in Section 3.4) also act as the role of testing images.

#### 4.1.2. Compared Methods

Seven representative multimodal image fusion methods are selected for performance comparison: the dual-tree complex wavelet transform- (DTCWT-) based method [3], the curvelet transform- (CVT-) based method [4], the non-subsampled contourlet transform- (NSCT-) based method [5], the sparse representation- (SR-) based method [9], the convolutional neural network- (CNN-) based method [36], the sharp frequency localization contourlet transform- (SFL-CT-) based method [23], and the complex shearlet transform- (CST-) based method [24]. The first three are based on popular multiscale transforms, and their parameters are set to the optimal values reported in an influential comparative study [42]. The fourth one is based on sparse representation via simultaneous orthogonal matching pursuit (SOMP) algorithm. The fifth one is a recently proposed deep learning- (DL-) based method, while the last two are the fusion methods specially designed for GFP and phase-contrast images. The parameters in these methods are all set to the default values for unbiased comparison.

#### 4.1.3. Objective Metrics

In [43], Liu et al. presented a comprehensive review of the objective evaluation metrics for image fusion and classified them into four categories: the information theory-based ones, the image feature-based ones, the image structural similarity-based ones, and the human perception-inspired ones. In this paper, to conduct an all-round objective assessment, one widely used metric is chosen from each category. The first one is the normalized mutual information (*Q*_MI_) [44] that measures the mutual dependence between the input images and the fused image. The second one is an image feature-based metric using phase congruency (*Q*_P_) [45]. This metric assesses the fusion quality through comparing the local cross correlation of corresponding feature maps of the input and fused images. The third one is Yang's metric (*Q*_Y_) [46], which evaluates the structural similarity between the input images and the fused one. The last one is proposed by Chen and Blum (*Q*_CB_) [47] based on human visual system (HVS) models. In addition, the visual information fidelity (VIF) measure [48] between the input phase-contrast image and the fused image is also employed for objective assessment. By characterizing the relationship between image information and visual quality, the VIF measure has been widely verified to be highly consistent with subjective evaluation. It is worth noting that the same measure between the GFP image and the fused image is not included. As reported in [23] (Table 1), the result on VIF measure between the GFP image and the fused image (the proposed method has the lowest score) is on the contrary with that of the VIF measure between the phase-contrast image and the fused image (the proposed method has the highest score). We also verify this point in our experiment. Specifically, we experimentally find that the result on VIF measure between the phase-contrast image and the fused image is highly consistent with other fusion metrics, while the situation for the GFP image is just on the contrary. One possible explanation for this issue is that most of the pixels or regions in the GFP image are dark (the intensity is zero), which is significantly different from the situations of the fused image or the phase-contrast image. Therefore, a higher VIF measure between the GFP image and the fused image may not indicate a better fusion result. Based on the above observations, only the VIF measure between the phase-contrast image and the fused image is used for evaluation in this work. For each of the above metrics, a higher score indicates a better performance.

### 4.2. Parameter Analysis

**Figure 5:**
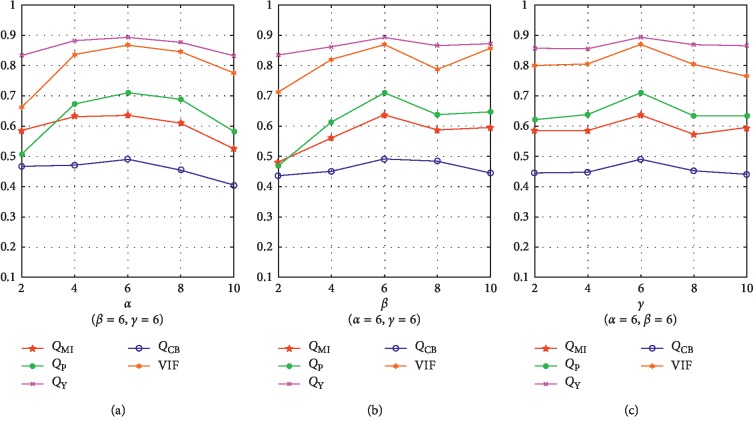
Influences of *α*, *β*, and *γ* on objective performance.

In this section, the impacts of three trade-off parameters *α*, *β*, and *γ* in our method are quantitatively studied via the objective fusion metrics. Based on a large quantity of experiments, we obtain an appropriate setting: *α*=6, *β*=6, and *γ*=6. As a popular approach for analysing the impacts of multiple parameters, the *controlling for a variable* is adopted to verify this point. The results are shown in Figure 5. Considering that it is practically difficult to show all the results that contain too many combinations, only one set of results is provided to exhibit the impact of each parameter, by fixing the other two as the well-performed values (this is a widely used manner in the study of image fusion [8, 38]). For each metric, the average score of 148 images is employed for evaluation in Figure 5. It is obvious that for each parameter, the best performances on all the five metrics are mostly obtained when its value is 6. Accordingly, these three free parameters are all set to 6 in our method.

### 4.3. Results and Discussion

**Figure 6:**
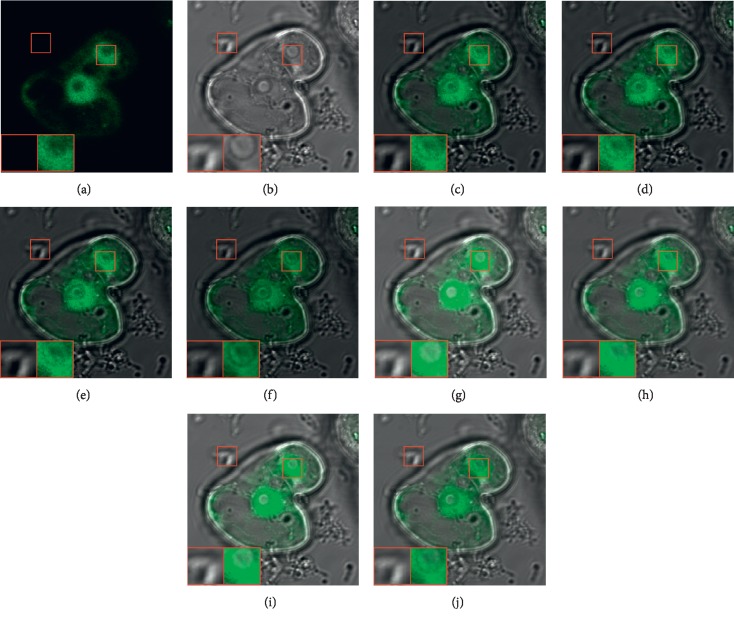
The first set of GFP and phase-contrast image fusion results. (a) GFP image. (b) Phase-contrast image. (c) DTCWT. (d) CVT. (e) NSCT. (f) SR. (g) CNN. (h) SFL-CT. (i) CST. (j) Proposed.

**Figure 7:**
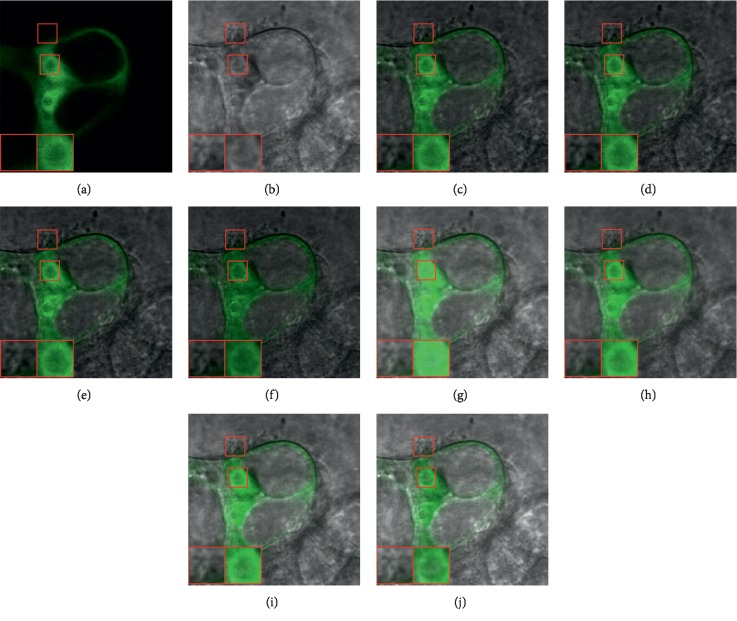
The second set of GFP and phase-contrast image fusion results. (a) GFP image. (b) Phase-contrast image. (c) DTCWT. (d) CVT. (e) NSCT. (f) SR. (g) CNN. (h) SFL-CT. (i) CST. (j) Proposed.

Figures 6 and 7 provide two sets of fusion results which include the input images and the fused images obtained by different methods. In each image, two representative regions are enlarged as close-ups for better comparison.

It can be seen that the DTCWT-based, CVT-based, NSCT-based, and SR-based methods can well capture the functional information from the GFP image and the spatial details from the phase-contrast image. However, these methods tend to lose a large amount of image energy from the phase-contrast image. As a result, the brightness of the fused images is obviously lower in comparison to the phase-contrast image, leading to undesirable visual artifact (see the first close-ups in Figures 6(b)–6(f) and 7(b)–7(f)).

For the CNN-based method, the image energy can be well preserved, but the functional information is not well tackled as the green regions are actually over emphasized when compared with the GFP input image. As a consequence, some structural details are concealed by the green regions (see the second close-ups in Figures 6(g) and 7(g)). The SFL-CT-based and CST-based methods achieve obvious improvement on this issue, but still suffer from this defect to a certain degree (see the second close-ups in Figures 6(h)-6(i) and 7(h)-7(i)).

The proposed method can achieve the highest visual quality among all the methods. On the one hand, the functional information from the GFP image is accurately preserved by method. On the other hand, the fused images of our method well inherit both the structural information and image energy from the phase-contrast image.

The objective assessment of different fusion methods on the above five metrics are listed in Table 1. For each method, the mean value (MV) and the standard deviation (SD) of each metric over 148 pairs of input images are reported. Moreover, the number of image pairs on which the corresponding method achieves the highest score is counted and termed as winning times (WT) in Table 1. The maximum mean value, minimum standard deviation, and maximum winning times among all the methods are indicated in bold. It can be seen that the proposed method clearly outperforms the DTCWT-based, CVT-based, NSCT-based, SR-based, CNN-based, and SFL-CT-based methods on all the five evaluation metrics. In comparison to the CST-based method that wins the first places on *Q*_Y_ and *Q*_CB_, our method owns obvious advantage on *Q*_MI_, *Q*_P_, and VIF, while achieving very close performance on *Q*_Y_ and *Q*_CB_. Besides, the proposed method obtains relatively small standard deviations on all the five metrics, which indicates that it can stably obtain high-quality fusion results.

Based on the above qualitative and quantitative comparisons, the proposed method exhibits clear advantages over the other seven methods. Moreover, the computational efficiency is sufficiently high for practical usage. Specifically, under the hardware environment consisting of an Intel Core i7-7820K CPU and a NVIDIA TITAN Xp GPU, it takes only about 0.06 seconds for our method to fuse two images of size 358 × 358 pixels. Since all the other methods are implemented in Matlab, their running time is not provided for comparison.

### 4.4. Influence of Network Architecture

In this section, we study the influence of network architecture on the fusion performance of the proposed method. Specifically, the impacts of the number of feature maps and the number of convolutional layers are studied. Firstly, two sets of experiments are conducted to investigate the influence of the number of feature maps, one of which is halving the number of the feature maps in the first four layers of the generator and the discriminator, and the other is doubling them. Secondly, to analyse the impact of the number of convolutional layers, we perform another two sets of experiments, one of which is removing the first layer of the generator and the fourth layer of the discriminator (both of them contain 256 feature maps), while the other is adding a convolutional layer with 512 feature maps into the generator before the first layer and into the discriminator after the fourth layer, respectively.

Table 2 lists the objective evaluation results of the above experiments, which are denoted by halved feature maps, doubled feature maps, reduced layers, and increased layers. The results of the original network architecture are also given as reference. For each approach, the mean value of each metric over 148 pairs of input images is reported. It can be seen that the proposed method can generally obtain better performance with more feature maps and convolutional layers. In particular, the number of feature maps has relatively more effect on the fusion performance in this task, in comparison to the number of convolutional layers. By taking the results given in Table 1 into consideration together, we can see that the proposed method with a slighter model (halved feature maps or reduced layers) is still competitive enough among all the fusion methods. A heavier model (doubled feature maps or increased layers) can provide some further improvement in terms of the original network architecture, but the extent is not significant. Considering the factors like memory consumption and computational efficiency, it is an appropriate choice to employ the network architectures described in Section 3 as the default settings.

### 4.5. Verification of the Overfitting Problem

As mentioned above, the proposed fusion method is essentially an unsupervised approach since there is no ground truth fused images used for training. Accordingly, the whole dataset can be employed for training and testing in the above experiments, without dividing it into training set and testing set. Although it is a reasonable manner to obtain the fusion results for all the images, the performance of the trained model on new testing data remains unknown.

To address this issue, we conduct a 5-fold cross validation to study if the proposed fusion model has the overfitting problem. Specifically, all the 148 pairs of images are randomly divided into five groups, with 30 pairs in the first four groups and 28 pairs in the last group. In each fold, four groups are employed as training data and the remaining one is used for testing. Therefore, each pair of images is employed for testing only once, and all the 148 fused images obtained in the testing process are used for objective evaluation.  Table 3 shows the objective assessment results of the five-fold cross validation experiment, along with the results of original training/testing manner for comparison. For each approach, the mean value of each metric over 148 pairs of input images is given. It is not surprising that the performance of the cross validation approach has a slight decrease when compared with that of the original manner. By referring to the performances of other fusion methods reported in Table 1, we can find that this decreasing extent is very small, which demonstrates that there is no obvious overfitting phenomenon and the proposed image fusion model has good practicality to new examples.

## 5. Conclusion and Future Work

In this paper, we propose a GFP and phase-contrast image fusion method based on generative adversarial networks. The fusion problem is addressed as an adversarial game between a generator and a discriminator by carefully considering the characteristics of different input images. Experimental results demonstrate that the proposed method can simultaneously extract the functional information from the GFP image and the structural information from the phase-contrast image, leading to better performance than several existing methods in terms of both visual quality and objective assessment. The proposed fusion framework is of high generality to functional and structural image fusion problems. In the future, we will study its feasibility in multimodal medical image fusion issues such as magnetic resonance (MR) and positron emission tomography (PET) image fusion.

## Figures and Tables

**Algorithm 1:**
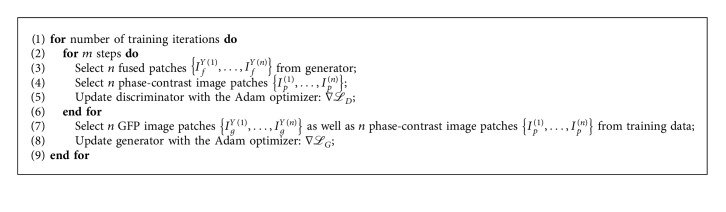
The procedure of network training in our method.

**Table 1:** Objective evaluation of different fusion methods.

Metric	Statistics	DTCWT	CVT	NSCT	SR	CNN	SFL-CT	CST	Proposed
*Q*_MI_	MV	0.2916	0.2725	0.3070	0.3877	0.4233	0.5142	0.5396	**0.6362**
	SD	0.0555	0.0493	0.0578	0.0461	0.1647	2.5361	25.4793	**0.0438**
	WT	0	0	0	0	16	9	12	**111**

*Q*_P_	MV	0.4374	0.4076	0.5011	0.6056	0.5218	0.5303	0.5597	**0.7098**
	SD	0.1645	0.1550	0.1616	0.1382	0.1881	0.1799	0.1750	**0.0697**
	WT	0	0	0	3	18	1	11	**115**

*Q*_Y_	MV	0.7185	0.7066	0.7372	0.7087	0.8570	0.8520	**0.8938**	0.8925
	SD	0.0743	0.0739	0.0701	0.0746	0.1298	0.1001	0.1060	**0.0669**
	WT	0	0	2	0	16	0	**75**	57

*Q*_CB_	MV	0.4540	0.4541	0.4634	0.4491	0.4646	0.4885	**0.4991**	0.4908
	SD	0.1045	**0.1030**	0.1081	0.1095	0.1350	0.1363	0.1377	0.1275
	WT	0	8	11	0	8	5	**69**	47

VIF	MV	0.6697	0.6623	0.6968	0.6926	0.7368	0.6785	0.7705	**0.8682**
	SD	0.1362	0.1343	0.1323	**0.0869**	0.1678	0.1147	0.1290	0.1236
	WT	0	0	0	0	29	0	29	**90**

The abbreviations MV, SD, and WT stand for mean value, standard deviation, and winning times, respectively.

**Table 2:** Objective evaluation results of the proposed method using different network architectures.

Metric	Halved feature maps	Doubled feature maps	Reduced layers	Increased layers	Original architecture
*Q*_MI_	0.5633	0.6471	0.5990	0.6395	0.6362
*Q*_P_	0.6015	0.7133	0.6319	0.7109	0.7098
*Q*_Y_	0.8564	0.8943	0.8664	0.8899	0.8925
*Q*_CB_	0.4748	0.4941	0.4841	0.4906	0.4908
VIF	0.7855	0.8728	0.8033	0.8696	0.8682

**Table 3:** Objective evaluation results for the verification of the overfitting problem.

Metric	5-Fold cross validation	Original training manner
*Q*_MI_	0.6135	0.6362
*Q*_P_	0.6678	0.7098
*Q*_Y_	0.8634	0.8925
*Q*_CB_	0.4819	0.4908
VIF	0.8355	0.8682

## Data Availability

The data supporting this study are from previously reported studies and datasets, which have been cited. The dataset used in this research work is available at http://data.jic.ac.uk/Gfp/, released by the John Innes Centre.
